# Impacts of continuous and rotational cropping practices on soil chemical properties and microbial communities during peanut cultivation

**DOI:** 10.1038/s41598-022-06789-1

**Published:** 2022-02-17

**Authors:** Huying Li, Chaohui Li, Xin Song, Yue Liu, Qixiong Gao, Rui Zheng, Jintai Li, Pengcheng Zhang, Xunli Liu

**Affiliations:** 1 College of Forestry, Shandong Agricultural University, No. 61, Daizong Street, Taian, 271018 Shandong China; 2 State Forestry and Grassland Administration Key Laboratory of Silviculture in Downstream Areas of the Yellow River, Taian, 271018 China

**Keywords:** Ecology, Microbiology

## Abstract

Long-term monocultures have severely inhibited the cultivation of Chinese peanut (*Arachis hypogaea* L.). In this study, the effects of continuous cropping on soil chemical properties and microbial communities were investigated in peanut fields that had been in crop rotation for 10 years and in monoculture for 10 years. The results found that long-term monoculture increased the activities of available potassium, available phosphorus, available nitrogen, soil organic matter, urease, acid phosphatase and catalase; while decreasing the activity of catalase. The diversity and abundance of soil bacteria and fungi is higher under continuous peanut cultivation. At the genus level, the relative abundance of potentially beneficial microflora genera was higher in the rhizosphere soil of rotational cropping than in continuous cropping, while the opposite was true for the relative abundance of potentially pathogenic fungal genera. Principal coordinates and cluster analysis indicated that continuous cropping altered the structure of the microbial community. The results of the functional predictions showed significant differences in the functioning of the rhizosphere microbial community between continuous and rotational cropping. In conclusion, long-term continuous cropping changed the chemical properties of the soil, altered the structure and function of the soil bacterial and fungal communities in peanut rhizosphere, which to some extent reduced the relative abundance of potentially beneficial microbial genera and increased the relative abundance of potentially pathogenic fungal genera, thus increasing the potential risk of soil-borne diseases and reducing the yield and quality of peanut. Therefore, in the actual production process, attention should be paid not only to the application of chemical fertilizers, but also to crop rotation and the application of microbial fertilizers.

## Introduction

The economic crop peanut (*Arachis hypogaea* L.) is a major source of oil; it originated in South America and has been cultivated since 350 BCE^1^. China is a major producer of peanuts, with approximately 5 million hectares planted annually, accounting for approximately 60–85% of the nation’s dry farming area^2,3^. The production scale of peanuts has continued to expand, and peanut production areas in China are now relatively concentrated. However, long-term consecutive monoculture problems are among the key factors currently affecting the productivity and quality of peanuts in China^4^. Monoculture is common in agricultural planting, but it causes problems such as reduced crop yield, nutritional disorder, soil microbiological deterioration, autotoxin accumulation, and the aggravation of pests and diseases^5–9^. After investigation, it was found that the yield of the sample plots selected for this study was about 3450 kg ha^−1^ for continuous cropping and 3900 kg ha^−1^ for rotational cropping. The yield of peanut under long-term monoculture was reduced by about 11%.

Previous research into continuous cropping has mainly focused on nutritional disorders and autotoxicity caused by allelochemicals in root exudates^10–12^. However, recent research has found that the rhizosphere is rich in microorganisms, which have been referred to as the second genome of plants; these microorganisms are crucial to plant health and crop yield^13^. Wu et al*.* found that during the growth and continuous cropping of *Rehmannia glutinosa*, the number of bacterial, and fungal populations in rhizosphere soil changed significantly, which destroyed the balance between beneficial and harmful microorganisms^14^. Galazka et al*.* found that soil enzyme activity and the total number of bacteria and actinomycetes increased significantly in soil following continuous corn cropping^15^. Wu et al*.* found that replanting diseases regarding *Pistacia chinensis* were closely related to changes in the structure and potential functions of the rhizosphere bacterial community^16^. Although reports have suggested that plant-microbial community interactions play a vital role in crop health, however, few reports have simultaneously researched the effects of continuous cropping on the structure and potential function of the bacterial and fungal communities of peanut rhizosphere soil. In addition, multiple studies have shown that changes in residential soil microflora are related to changes in soil physical and chemical properties, soil enzyme activities, plant species, and the type of soil environment^17–19^. Therefore, herein, it was hypothesized that long-term consecutive monoculture in peanuts may directly influence the soil microbial community and its chemical properties and may further negatively affect plant growth.

Rhizosphere soils from continuous cropping and rotation peanut fields were sampled in this study. The physical and chemical characteristics of the total soil organic matter (SOM), available nitrogen (AN), available phosphorus (AP), available potassium (AK), soil bacteria structures, and fungal community structures of both types of fields were analyzed and compared. The bacterial and fungal communities and physical and chemical characteristics of soil under the two different peanut planting modes were studied. The relationship between the rhizosphere microbial community structure and the obstacles to continuous peanut cropping was then discussed, providing a theoretical basis for its control.

## Results

### Bacterial and fungal community composition

**Figure 1:**
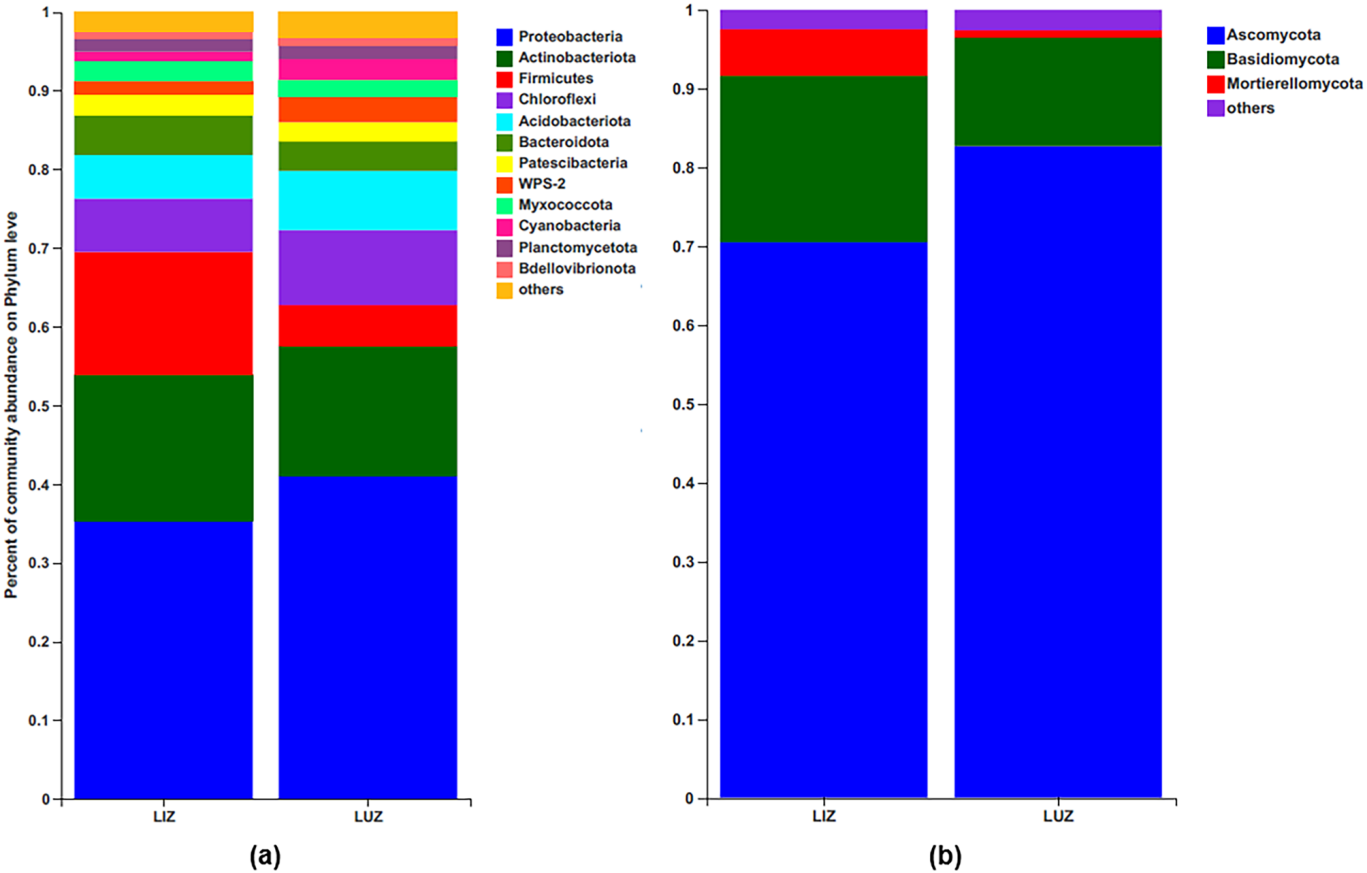
Relative abundance of main bacterial (**a**) and fungal (**b**) phylum in LUZ and LIZ rhizosphere soil. *LUZ* rotation, *LIZ* continuous cropping.

After filtering reads according to basic quality control and the removal of a single OTU, 758,780 sequences were obtained from 12 samples, including 3,611 bacterial OTUs. The lengths of high-quality sequences per sample ranged from 202 to 516 bp. The bacterial OTUs were assigned to 29 phyla, 85 classes, and 685 genera. The dominant phyla (average relative abundance > 1%) across all samples were Proteobacteria (38.13%), Actinobacteria (17.56%), Firmicutes (10.46%), Chloroflexi (8.12%), Acidobacteria (6.52%), Bacteroidetes (4.43%), Patescibacteria (2.52%), WPS-2 (2.51%), Myxococcota (2.30%), Cyanobacteria (1.93%), and Planctomycetota (1.65%; Fig. 1a).

After filtering the reads as indicated above, 868,491 fungal sequences were obtained from 12 samples, and sequences were clustered into 760 OTUs. The lengths of these high-quality sequences per sample ranged from 141 to 511 bp. The fungal OTUs were assigned to 12 different phyla, 36 classes, and 271 genera. Ascomycota accounted for 76.60% of all fungal OTUs. The other two dominant phyla were Basidiomycota (17.44%) and Mortierellomycota (3.45%) (Fig. 1b).

The relative abundances of different phyla in monoculture (LIZ) and rotation (LUZ) soils were compared (Fig. 1). As shown in Fig. 1a, LIZ resulted in a significantly higher relative abundance of Firmicutes and a significantly lower relative abundance of WPS-2 in the dominant phyla (P < 0.05), compared to LUZ (Fig. S1). Although there were differences between the other dominant bacteria phyla, these differences were not significant. The relative abundance of Ascomycetes in the dominant fungal phyla (top three) was significantly lower in LIZ, while the relative abundance of Mortierellomycota was significantly higher (P < 0.05), compared to LUZ (Figs. 1b, S2).

**Figure 2:**
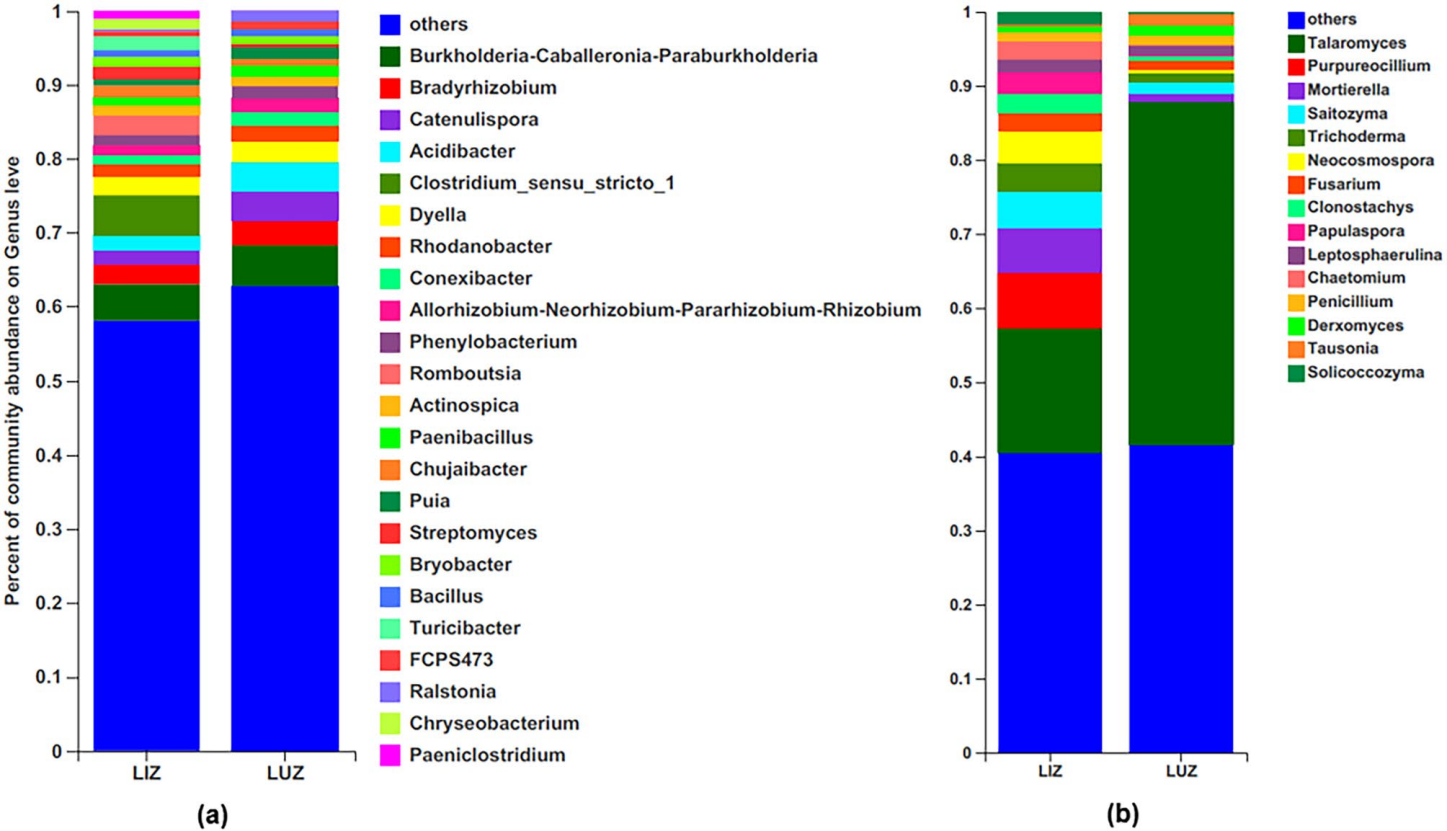
Relative abundance of main bacterial (**a**) and fungal (**b**) genus in LUZ and LIZ rhizosphere soil. *LUZ* rotation, *LIZ* continuous cropping.

At the genus level, the dominant bacterial genus was *Burkholderia–Caballeronia–Paraburkholderia*. The bacterial genera that showed significant differences were *Acidibacter*, *Clostridium_Sensu_Stricto_1*, *Turicibacter*,* Puia*, *Romboutsia*, *Streptomyces*, *Bryobacter*, *Paeniclostridium* and *Ralstonia* (Figs. 2, S3). Regarding fungi, the dominant genus was *Talaromyces*. The genera with significant differences between monocillium and rotated rhizosphere soils were as follows: *Talaromyces*, *Chaetomium*, *Mortoerella*, *Neocosmospora*, *Solicoccozyma*, and *Papulaspora*(P < 0.05) (Figs. 2, S4).

### Bacterial and fungal α-diversity

As shown in Table 1, the coverage rate was above 98% for all samples, which indicates that the depth of sequencing met the needs of the experiment. The Chao1 and ACE indices of bacterial and fungal communities were significantly higher for LIZ than for LUZ (P < 0.05). The Shannon index of bacteria showed no significant difference between the two tillage practices, whereas the Shannon index of fungi was significantly higher in LIZ. These results showed that long-term continuous cropping resulted in relatively high abundance and diversity of fungi and high abundance of bacteria without significant changes in diversity in peanut fields.

**Table 1:** Bacterial and fungal alpha-diversity indexes of soil from the two peanut fields.

Microbial community	Peanut fields	ACE	Chao 1	Shannon	Coverage
Bacteria	LUZ	1974.44 ± 97.91b	1985.30 ± 99.54b	5.68 ± 0.172a	99.09 ± 0.07a
	LIZ	2451.16 ± 107.07a	2461.10 ± 106.88a	5.74 ± 0.27a	98.79 ± 0.05b
Fungi	LUZ	243.36 ± 29.92b	243.73 ± 27.67b	2.27 ± 0.48b	99.94 ± 0.01a
	LIZ	394.95 ± 53.40a	392.12 ± 53.15a	3.11 ± 0.34a	99.89 ± 0.01b

Different letters within the same column indicate significant difference between treatments tested by one-way ANOVA (P < 0.05). Values are the means ± SE.

### Bacterial and fungal β-diversity

**Figure 3:**
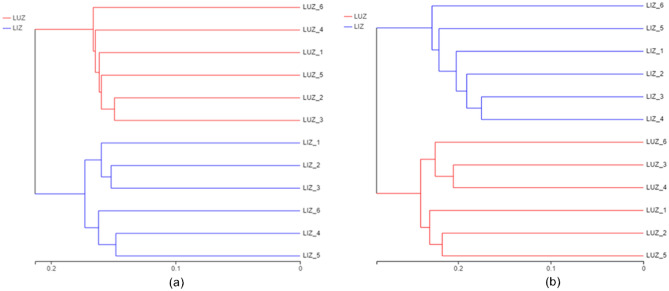
Unweighted pair-group method with arithmetic mean (UPGMA) dendrogram of bacterial (**a**) and fungal (**b**) communities based on unweighted_unifrac distance. *LUZ* rotation, *LIZ* continuous cropping.

In the cluster analysis of bacteria (Fig. 3a), all samples were grouped into two, among which samples from the same farming method were grouped. Tillage methods appeared to have some influence on the bacterial community structure of the soil in the rhizosphere of peanuts. Similar results were also observed for fungi (Fig. 3b).

**Figure 4:**
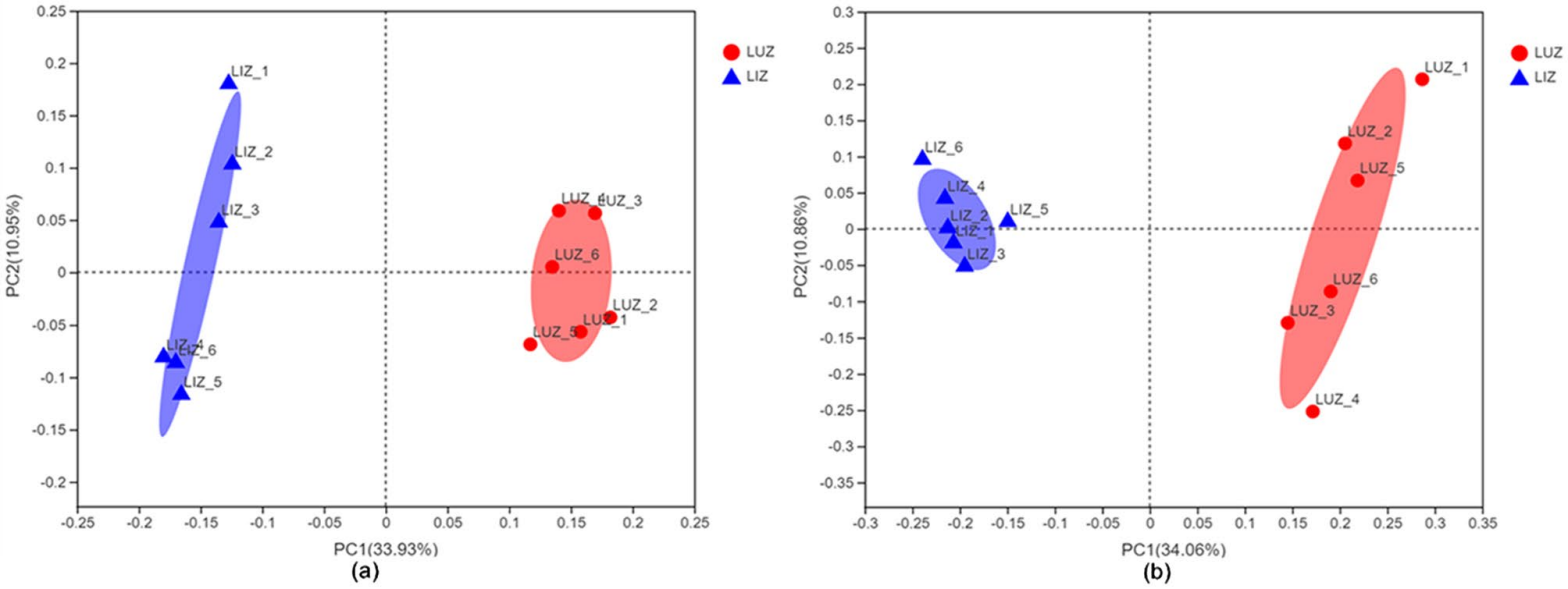
Principal coordinate analysis (PCoA) of bacterial (**a**) and fungal (**b**) communities based on unweighted_unifrac distance. *LUZ* rotation, *LIZ* continuous cropping.

PCoA of bacteria and fungi in the rhizosphere soils of LUZ and LIZ revealed that the first two axes explained 44.88% of the variation in the bacterial data and 44.92% of the variation in the fungal data (Fig. 4). These results indicated that there were significant differences in the composition and structure of the bacterial and fungal communities between LUZ and LIZ. PCoA and cluster analysis revealed that the microbial community structure of peanut rhizosphere soil was affected by the tillage method used.

### Soil chemical properties and enzyme activity on bacterial or fungal taxa

The chemical properties of rhizosphere soil under the different tillage methods are shown in Table 2. The activities of AN, AK, AP, SOM, urease, phosphatase, and sucrase in soil were significantly higher in LIZ than in LUZ, while catalase activity in rotation soil was slightly higher than that in monocropping.

**Table 2:** Soil characteristics and enzyme activity from the two peanut fields.

	LUZ	LIZ
Alkaline-hydrolysis nitrogen (mg/kg)	36.12b	55.81a
Available phosphorus (μg/kg)	15.76b	78.40a
Available potassium (mg/kg)	95.57b	261.63a
Soil organic matter (g/kg)	4.67b	8.38a
Urease (mg/g)	5.33b	7.96a
Phosphatase (mg/g)	5.02b	8.33a
Sucrase (mg/g)	0.34b	0.55a
Catalase (mg/g)	0.26a	0.24a

Different letters within the same column indicate significant difference between treatments tested by one-way ANOVA (P < 0.05).

**Figure 5:**
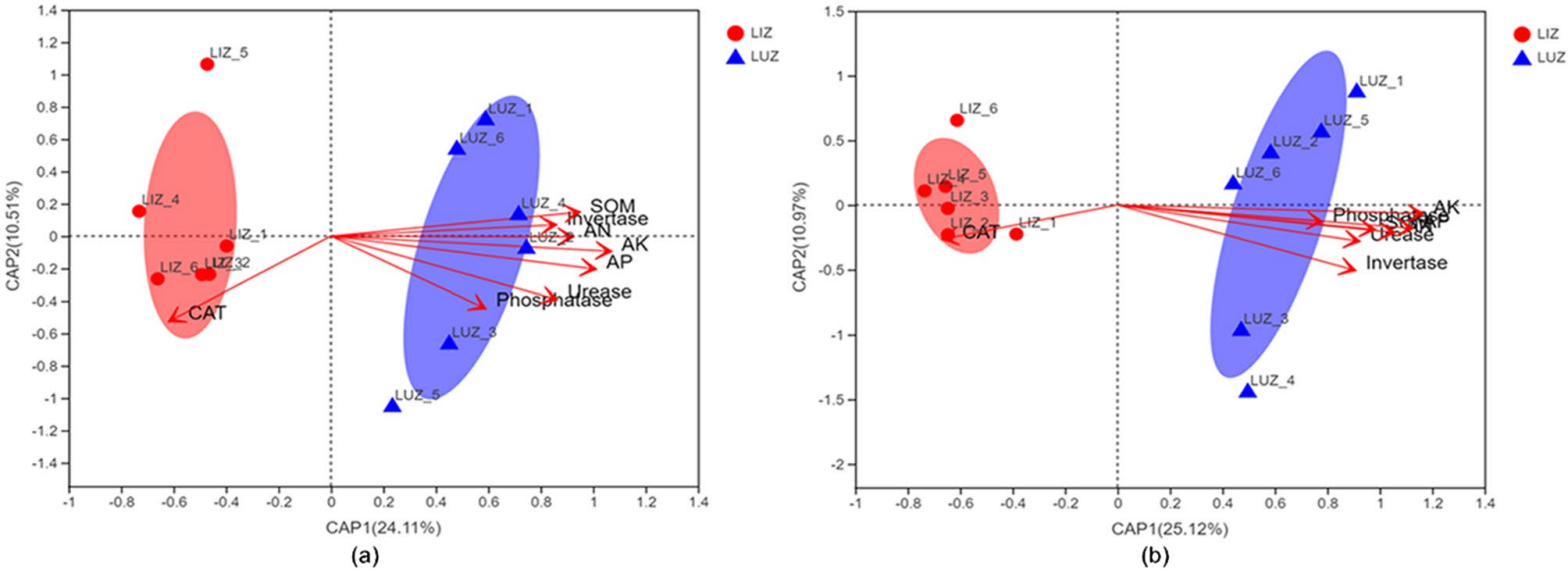
Distance-based redundancy analysis (db-RDA) based on the relative abundances of bacterial (**a**) and fungal (**b**) genus and environmental variables. *SOM* soil organic matter, *AN* available nitrogen, *AP* available phosphorus, *AK* available potassium, *LUZ* rotation, *LIZ* continuous cropping.

Distance-based redundancy analysis (db-RDA) based on the relative abundances of bacterial and fungal genus and environmental variables. Db-RDA showed that the first and second CAP components accounted for 34.62 and 36.09% of the total bacterial and fungal variations, respectively (Fig. 5). For bacteria and fungi, the LIZ treatment was separated from the LUZ treatment by the first component (CAP1). Soil AK was the most important factor in shifting the bacterial and fungal community structures.

### Prediction of microbial community function

**Figure 6:**
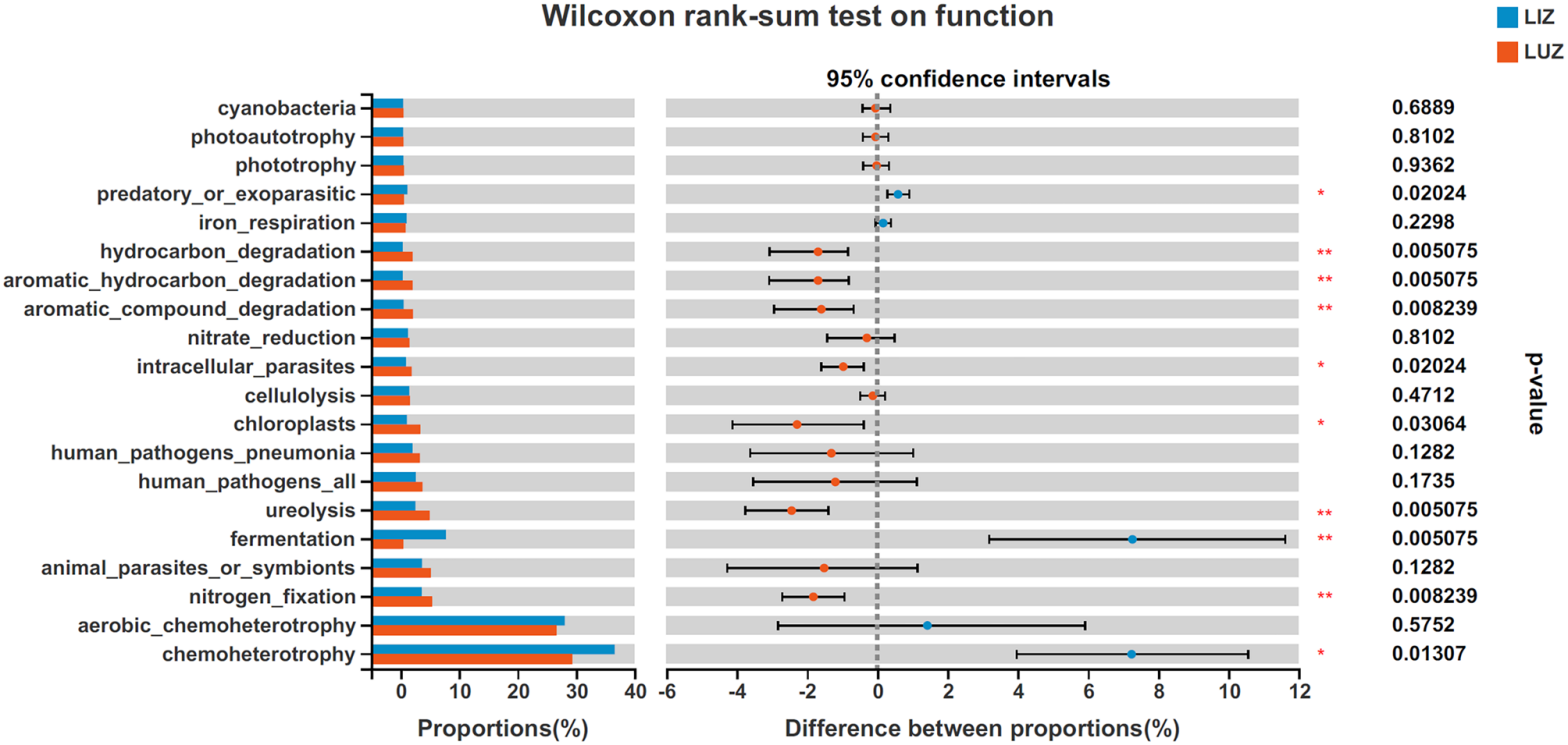
Wilcoxon rank-sum test for functional prediction of the top 20 bacterial genera. The Y-axis indicate the function name and the X-axis indicate the percentage value of the abundance of a particular function for that sample, with different colours indicating different groupings. P values are on the far right, *0.01 < P ≤ 0.05, **0.001 < P ≤ 0.01.

In order to study the bacterial function of the different samples, functional prediction analysis was carried out using FAPROTAX software. The predicted results showed that the main functions of the bacterial community were chemoheterotrophy, aerobic_chemoheterotrophy, nitrogen_fixation, animal_parasites_or_symbionts, fermentation and ureolysis (Fig. 6). Nitrogen_fixation, ureolysis, chloroplasts, intracellular_parasites, aromatic_compound_degradation, aromatic_hydrocarbon_degradation and hydrocarbon_degradation functions were significantly higher in the percentage of rotational cropped fields than in the percentage of continuous cropped fields, while the opposite was true for chemoheterotrophic, fermentation and predatory_or_exoparasitic. The figure indicated that the relative abundance of functional groups associated with nitrogen and carbon cycling was mostly higher in rotational cropped fields than in continuous cropped fields.

**Figure 7:**
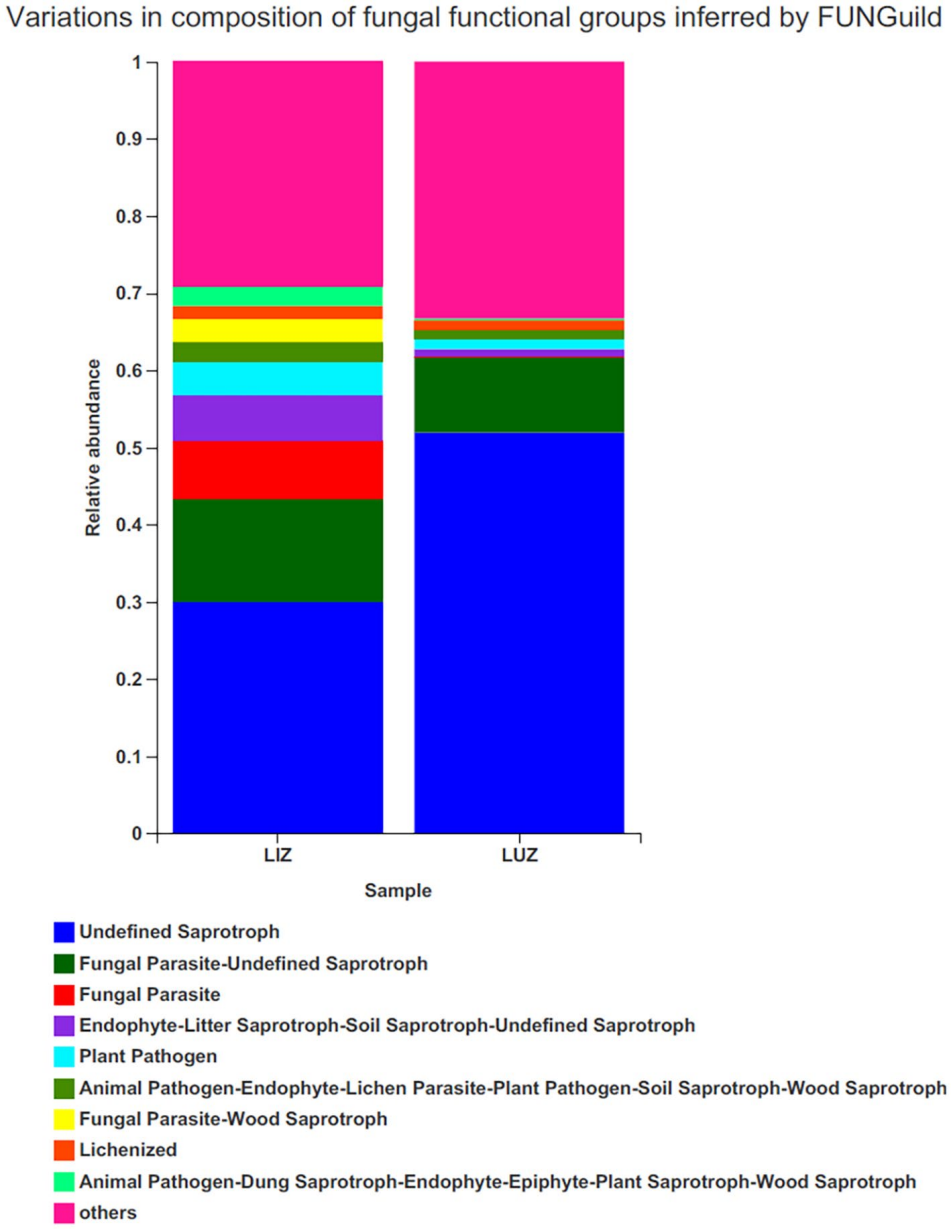
Compositions of fungal functional groups (guild) inferred from FUNGuild. The Y-axis is the proportion of Guild’s abundance in different samples, and the X-axis is the name of different samples.

FUNGuild was used to predict the trophic and functional groups of fungal communities for the different treatments (Fig. 7). FUNGuild detected all three main trophic modes and 60 guilds. There were nine trophic patterns in the rhizosphere fungi, of which the most abundant was Undefined Saprotroph (41%), followed by Fungal Parasite-Undefined Saprotroph(11.5%), Fungal Parasite (3.8%), Endophyte-Litter Saprotroph-Soil Saprotroph-Undefined Saprotroph (3.4%), Plant Pathogen (2.8%), Animal Pathogen-Endophyte-Lichen Parasite-Plant Pathogen-Soil Saprotroph-Wood Saprotroph (1.8%), Fungal Parasite-Wood Saprotroph (1.4%), Lichenized (1.4%) and Animal Pathogen-Dung Saprotroph-Endophyte-Epiphyte-Plant Saprotroph-Wood Saprotroph (1.3%). The results showed that the relative abundance of Saprotroph, Fungal Parasite, Plant Pathogen and Animal Pathogen was higher in the continuous crop sample plots than in the rotation sample plots.

## Discussion

Long-term continuous monoculture can lead to a severe decrease in peanut yield, as shown in previous studies^4,20^. Here, PCoA and hierarchical clustering analysis showed that there were significant differences in bacterial and fungal community structures between LUZ and LIZ. There were also differences in microbial richness and diversity in the rhizosphere soil between LUZ and LIZ. The soil bacterial richness and diversity index showed an increasing trend after continuous cropping, which was consistent with the findings of previous studies into continuous cropping for *Andrographis paniculata*, *Vanilla planifolia*, and *Rehmannia glutinosa*, contrary to findings regarding coffee, *American ginseng*, and *Panax notoginseng*^21–26^. Here, the fungal richness and diversity index were found to be higher under continuous cropping than under rotation, consistent with previously reported results for sweet potato (*Ipomoea batatas*), *Pseudostellaria heterophylla*, and alfalfa (*Medicago sativa*) under continuous cropping conditions, contrary to results regarding *Atractylodes macrocephala*^27–30^. The above results indicated that continuous cropping can had certain effects on the structure and composition of soil microbial communities and that differences in microbial diversity and richness in different studies may be related to not only long-term monoculture but also environmental conditions, plant types, fertilization, and other factors.

Here, changes in bacterial and fungal communities in the rhizosphere soil of peanuts grown under continuous cropping were identified through taxonomic analysis. For bacteria, the relative abundances of Proteobacteria, Chloroflexi, Acidobacteria, and WPS-2 were lower for LIZ than for LUZ, while the relative abundances of Actinobacteria and Firmicutes were higher. Proteobacteria was shown to be dominating the bacterial community and soil types in different geographic regions^31^. This phylum is the most dynamic taxon associated with rhizoctonia disease suppression, and play a key role in phylogenetic, ecological and pathogenic values and participates in energy metabolism, such as the oxidation of organic and inorganic compounds and obtaining energy from light^32,33^. In this study, Proteobacteria is the most abundant phylum in the rhizosphere soil of peanut monoculture. This result was consistent with the research result that proteobacteria were the most dominant in the continuous cropping soil of pineapple^34^, strawberry^35^ and coffee^24^. The relative abundance of Proteobacteria increased after monoculture of potato^36^ and black pepper^37^, while decreased in sugar beet^5^ and *Panax notoginseng*^38^. Many factors affect the composition of rhizosphere bacteria, among which root exudates and plant species play key roles. This results in the presence of different plant genotype-specific community structures even in the same soil^21^. Acidobacteria are the most abundant bacterial phylum in soils with meager resource utilization^39^. Long-term continuous cropping can lead to the decrease of soil nutrients, which may be the cause of the increase in the relative abundance of Acidobacteria. Regarding fungi, Ascomycota and Basidiomycota were the dominant phyla of soil fungi in the rhizosphere soil after peanut continuous cropping, which was consistent with previous studies^27,40^. After continuous cropping, the relative abundance of Ascomycota decreased, while the relative abundance of Basidiomycota and Mortierellomycota increased. Ascomycetes play an important role in the degradation of organic matter in rhizosphere soil, and the decrease of this fungi phylum content may affect soil fertility^41^.

At the genus level, the relative abundances of beneficial bacterial genera, such as *Burkholderia*, *Bradyrhizobium*, *Rhodanobacter*, *Dyella*, and *Bacillus*, were lower for LIZ than for LUZ. Some *Burkholderia* bacteria promote plant growth and antagonize nematodes^42^. Peanuts mainly form a symbiotic relationship with different *Bradyrhizobium* species, which fix atmospheric nitrogen into binding nitrogen (ammonia), which can then be used by host plants^42–44^. *Dyella* species have been reported to have a variety of plant growth-promoting properties, while *Bacillus* and *Rhodanobacter* have biological control activities^45–48^. Here, LIZ rhizosphere soil also exhibited significant differences in its fungal community at the genus level, compared with that of LUZ. The relative abundance of *Talaromyces* was significantly higher in LIZ than LUZ. *Talaromyces* species have antagonistic fungal functions on species such as *Cylindrocarpon destructans*, *Fusarium oxysporum*, *Rhizoctonia solani*, *Sclerotinia nivalis*, *Botrytis cinerea*, and *Phytophthora capsica*^49^. The relative abundance of pathogens such as *Fusarium*, *Penicillium*, *Gibberella* and *Colletotrichum* in LUZ rhizosphere soils was lower than in LIZ, and *Athelia* were not detected in the LUZ rhizosphere soil, while the relative abundance of *Athelia* in the LIZ field was 0.0916% (Table S1). *Penicillium* is a toxin-producing genus that can cause fruit, vegetable, and meat rots, as well as citrus penicillium^50^. *Fusarium*, which can cause plant rots, stem rot, flower rot and spike rot^51^. *Gibberella* is often used as the sexual stage of Fusarium fungi, many of which can cause devastating plant diseases and produce specific toxins or active metabolites that are toxic to humans and animals^52^. The prediction of fungal function revealed that the relative abundance of pathotroph fungi in the rhizosphere soil was higher in LIZ than in LUZ. The long-term continuous cropping of peanuts resulted in fewer beneficial fungi and more pathogenic fungi, which might be one of the important factors for peanut production, which is consistent with the results of existing studies^53^.

Table 2 shows that the contents of SOM, AN, AP, and AK were all higher in LIZ than in LUZ, which may have arisen from the overuse of inorganic fertilizers^36,37^ or the insufficient utilization of nutrients in the soil by peanuts after a long period of monoculture^54^. The study on cucumber continuous cropping showed that the soil nutrient contents of OM, AN, AK and AP were the highest in the seventh crop, while the biomass of cucumber was the lowest in the ninth crop. However, the soil nutrient content decreased and the biomass of cucumber increased^55^. Shao et al*.* also found that as the continuous cropping of peanuts progressed, the contents of AN, AP, AK, and organic carbon increased significantly^56^. Differences in nutrient content may occur due to significant differences in nutrient absorption and utilization during the continuous cropping of peanuts^24^.

The activities of urease, phosphatase, and invertase were significantly higher in LIZ than in LUZ. The plant type, continuous planting time, and fertilization regime can have different effects on the activities of various enzymes^57–60^. The volume of plant residue deep in the root system can also affect enzyme activity^61^. Galazka et al*.* suggested that enzyme activity is dependent not only on the type of plant but also on the volume of plant residue deep in the root system^15^. Therefore, the accumulation of plant residue during long-term peanut monoculture may have increased enzyme activity. Agomoh et al*.* found that increasing crop diversity through wheat rotation increased yields but reduced soil health^62^. These results indicate that the effects of the observed changes in enzyme activity might have been less influential than the effects of the change in microbial community structure regarding obstacles to continuous cropping.

The PCoA results showed that the bacterial and fungal communities in the soil in LIZ and LUZ were different. This phenomenon has also been observed in plants such as *Andrographis paniculata*, Pineapple (*Ananas comosus*), and soybean (*Glycine max*)^21,34,63^. Combined with the RDA results, the significant changes observed in the bacterial and fungal community structures might have been caused by changes in the soil's chemical properties. Investigating the correlation between microbial community diversity and soil environmental factors helps us to better understand the mechanisms of succession barriers. In our study, RDA results suggest that many soil properties may influence microbial community structure. We found that the structure of bacterial and fungal communities was mainly driven by AK and AP. Previous studies have shown that environmental variables affects the structure of microbial communities. For example, the Basidiomycota in the rhizosphere soil of coffee with 57 years of continuous crop was closely related to the content of AP^24^. In a research on long-term continuous cropping in strawberries, environmental variables were found to be positively correlated with non-continuously cropped strawberry rhizosphere soil and negatively correlated with continuously cropped strawberry rhizosphere soil^35^. Notably, changes in microbial community structure during long-term peanut continuous cropping may also be caused by the long-term effects of peanut residues or root exudates^24^.

Soil microbial biomass is an important indicator of soil quality, which reflects the processes of nutrient transfer and energy cycling^64^. The nitrogen cycle consists of complex interaction pathways including assimilative nitrate reduction, isochemical nitrate reduction, denitrification, nitrogen fixation, nitrification and anaerobic ammonia oxidation. We hypothesized, based on the predicted results of microbial functions, that reduced nitrogen fixation (significant difference) due to continuous cropping leads to less nitrogen being taken up by plants from the atmosphere and that reduced nitrate reduction (no significant difference) leads to more ammonia being produced intracellularly, and that the accumulated nitrite and ammonia may then become toxins, leading to a reduction in biomass (Fig. 6). Another cause of succession disorders may be related to the increase in plant pathogenic fungi (Fig. 7).

## Conclusion

These results provide new insights into changes in peanut rhizosphere microbial community structure based on high-throughput analysis of rhizosphere soils in continuous cropping and crop rotation. In summary, the main causes of growth inhibition and yield decline in peanut under long-term continuous cropping may be related to changes in soil microbial structure and potential functions, and the relative abundance of pathogenic microorganisms such as *Fusarium* and *Athelia* was higher in continuous rhizosphere soils than in rotational cropping sample plot (Table S1). Future research is needed to further elucidate the triangular relationship between soil characteristics, potential beneficial microbiota and peanut growth. This research provides a theoretical basis for developing sustainable agricultural practices, improving soil microbial activity and promoting peanut production in China.

## Materials and methods

### Ethics

The samples in the study were collected on private land where the owner allowed the study. The experimental materials did not involve any humans or animals. All methods comply with local and national guidelines.

### Sampling site

Soil samples were collected from Yishui County, Linyi City, Shandong Province, China (35°40′ 05′ N, 118°42 ′45′ E). The altitude of the study area is 160 m, the annual average temperature is 12.3 °C, and the annual rainfall is 750–850 mm. Under continuous cropping conditions, peanuts were planted continuously for at least 10 years (LIZ). For the rotation fields, vegetables, maize, wheat and peanuts had been rotated for more than 10 years (LUZ), crop rotation sequence of rape (or turnip), peanut, winter wheat, with summer maize in alternate years. The amount of fertilizer applied was 600 kg ha^−1^ of compound fertilizer (N:P_2_O_5_:K_2_O = 16:13:16), 120 kg ha^−1^ of micronutrient fertilizer and 600 kg ha^−1^ of organic fertilizer. Six sample sites were randomly selected from each sample site as replicates. First, litter was removed from the ground, following which the peanut roots were carefully removed. These roots were shaken to remove loosely attached soil. Any soil that was still firmly attached to the roots was then collected to sample the rhizosphere soil. The samples were collected in September 2020. Twelve soil samples were placed in sterile plastic bags and shipped to the laboratory in ice boxes. A portion of each sample was stored at − 80 °C for deoxyribose nucleic acid (DNA) extraction, while the remainder were air-dried for analysis of soil properties.

### Soil DNA extraction and polymerase chain reaction amplification

Soil total DNA was extracted from 12 soil samples using a TIANamp Soil DNA Kit, according to the manufacturer’s instructions. Genomic DNA concentration and purity were measured using a NanoDropND-2000 spectrophotometer. DNA integrity was determined via agarose gel electrophoresis. Specific primers of the bacterial 16S ribosomal ribonucleic acid (rRNA) genes and partial internal transcribed spacer regions of fungi were used for amplification. The primer set 338 F (5′-ACTCCTACGGGAGGCAGCAG-3′) and 806 R (5′-GGACTACHVGGGTWTCTAAT-3′) was used to amplify the V3-V4 hypervariable region of the bacterial 16S rRNA gene. ITS1F (5′-CTTGGTCATTTAGAGGAAGTAA-3′) and ITS2 (5′-GCTGCGTTCTTCATCGATGC-3′) were selected to target the fungal ITS1 region. Polymerase chain reactions (PCR) were performed in 20 ul reactions using the following temperature program: 95 °C for 3 min, 27 cycles of 95 °C for 30 s, 55 °C for 30 s, and 72 °C for 45 s. A final step of 72 °C for 10 min is followed by a holding step at 4 °C. The DNA was then sequenced using the Illumina Miseq PE300 platform (Illumina, San Diego, USA) at Majorbio Bio-Pharm Technology Co., Ltd. (Shanghai, China).

### Bioinformatics and data analysis

After removing the adaptors and primer sequences, raw sequences were assembled for each sample according to the unique barcode using quantitative insights into microbial ecology (QIIME)^65^. Flash v. 1.2.7 was used to merge the split sequences of each sample^66^. The valid labels of all samples were clustered using Uparse software^67^. Valuable sequences were divided into operational taxonomic units (OTUs) using USEARCH software, with a 97% identity threshold as the cut-off point. For species annotation, representative bacterial sequences were matched against the SILVA database (version 132)^68^, and the fungal representative sequences were classified using the UNITE database (version 8.0)^69^. The Mothur software was used to analyze the alpha diversity. The R language was used for distance-based redundancy analysis (db-RDA), principal coordinate analysis (PCoA), and unweighted paired group algorithm clustering. The unweighted-unifrac index was used as the distance measure. The Functional Annotation of Prokaryotic Taxa database (FAPROTAX) was used to predict bacterial functional groups. FUNGuild (http://www.funguild.org/) database was used to annotate fungal functions.

### Sequence accession numbers

Sequences data were deposited in the NCBI Sequence Read Archive (SRA) database under the BioProject: PRJNA746121 (the accession number SRR15112779-SRR15112790 for bacteria and SRR15115453–SRR15115464 for fungi).

### Analysis of soil chemical properties

The chemical properties were determined according to the method described by Bao^70^. Briefly, AK was determined photometrically using a flame spectrophotometer. AP was detected through colorimetry using a spectrophotometer. SOM content was assayed using acidified potassium dichromate (K_2_Cr_2_O_7_–H_2_SO_4_). AN was determined using the alkaline hydrolysis diffusion method.

### Analysis of soil enzymes activity

Soil urease activity was determined using the phenyl disodium phosphate colorimetric method^71^. Catalase activity was determined using K_2_Cr_2_O_7_/acetic acid reagent colorimetry^72^. The 3,5-dinitrosalicylic acid colorimetric method was used to measure soil invertase activity^73^. Phosphatase activity was determined using p-nitrophenol colorimetry^74^.

### Statistical analysis

All experimental data were analyzed using Microsoft Excel 2019 and IBM SPSS 25.0. Statistical significance was set at *P* < 0.05.

## Supplementary Information

Supplementary Legends.

Supplementary Figure S1.

Supplementary Figure S2.

Supplementary Figure S3.

Supplementary Figure S4.

Supplementary Table S1.
