# Supplementary material for: Impacts of continuous and rotational cropping practices on soil chemical properties and microbial communities during peanut cultivation
Source: Sci Rep. 2022 Feb 17;12:2758. doi: 10.1038/s41598-022-06789-1 (PMC8854431; doi:10.1038/s41598-022-06789-1)
Supplement: Supplementary file 1 — Supplementary Legends. [file 41598_2022_6789_MOESM1_ESM.docx]

**Figure S1.** Wilcoxon rank-sum test bar plot on bacterial Phylum level. The X-axis represents different samples, with different coloured bars indicating different samples, and the Y-axis represents the average relative abundance of a species in different samples.

**Figure S2.** Wilcoxon rank-sum test bar plot on fungal Phylum level. The X-axis represents different samples, with different coloured bars indicating different samples, and the Y-axis represents the average relative abundance of a species in different samples.

**Figure S3.** Wilcoxon rank-sum test bar plot on bacterial genus level. The X-axis represents different samples, with different coloured bars indicating different samples, and the Y-axis represents the average relative abundance of a species in different samples.

**Figure S4.** Wilcoxon rank-sum test bar plot on fungal genus level. The X-axis represents different samples, with different coloured bars indicating different samples, and the Y-axis represents the average relative abundance of a species in different samples.
