# Supplementary figures and images for: Impacts of continuous and rotational cropping practices on soil chemical properties and microbial communities during peanut cultivation
Source: Sci Rep. 2022 Feb 17;12:2758. doi: 10.1038/s41598-022-06789-1 (PMC8854431; doi:10.1038/s41598-022-06789-1)

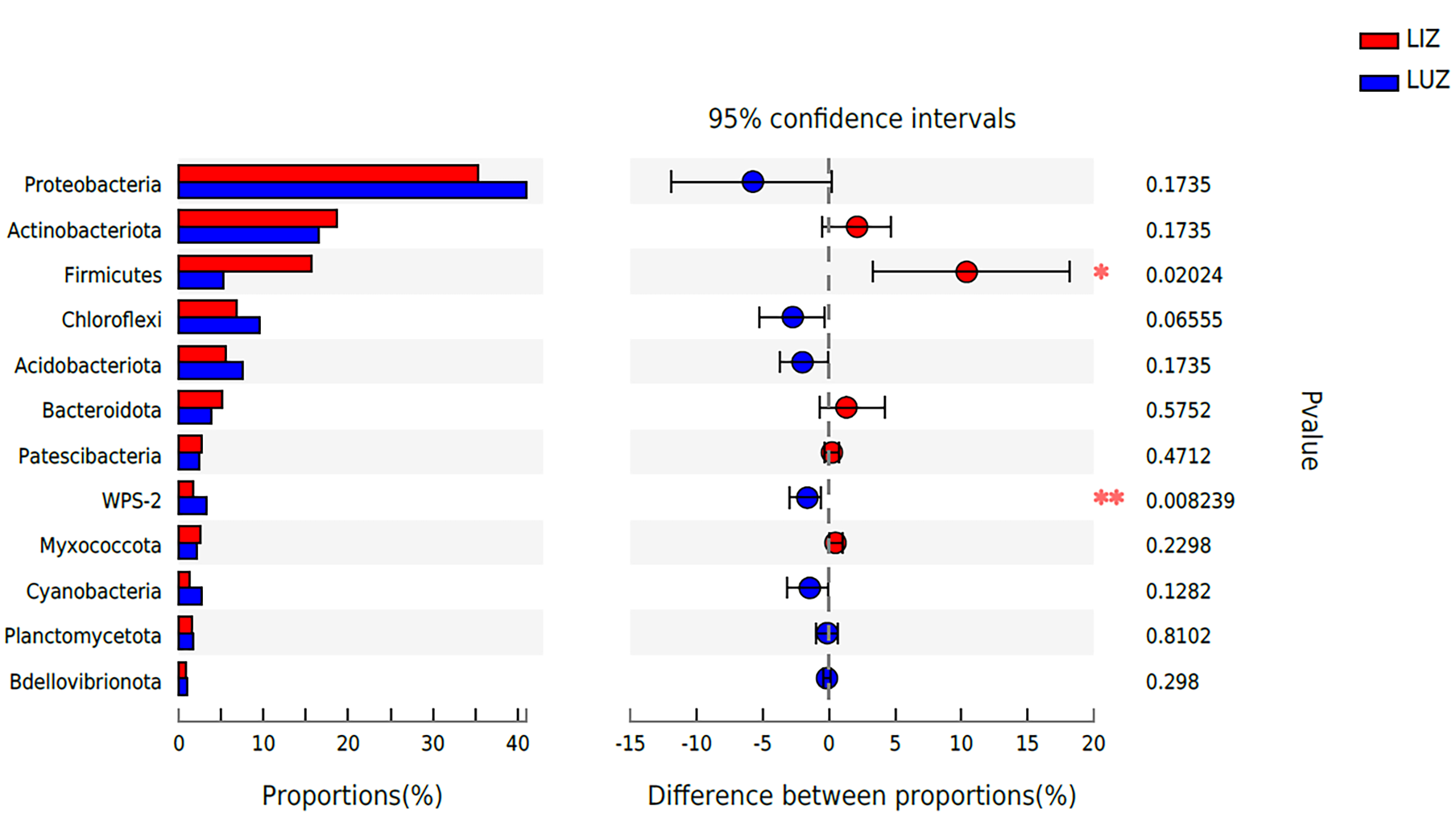

Supplement: Supplementary file 2 — Supplementary Figure S1. [file 41598_2022_6789_MOESM2_ESM.tif]

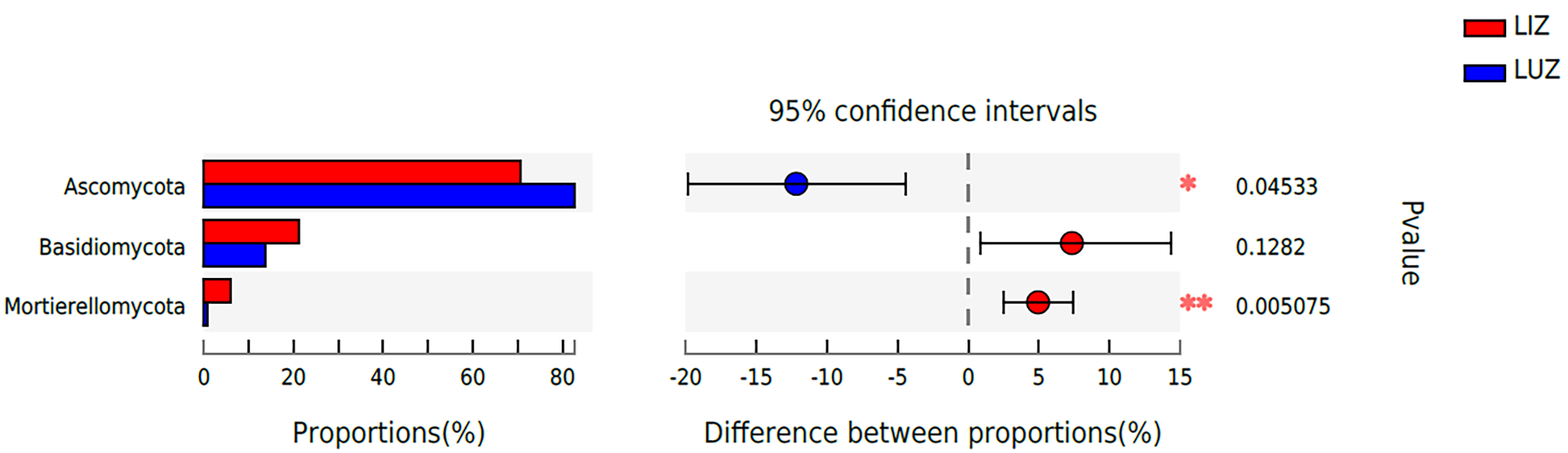

Supplement: Supplementary file 3 — Supplementary Figure S2. [file 41598_2022_6789_MOESM3_ESM.tif]

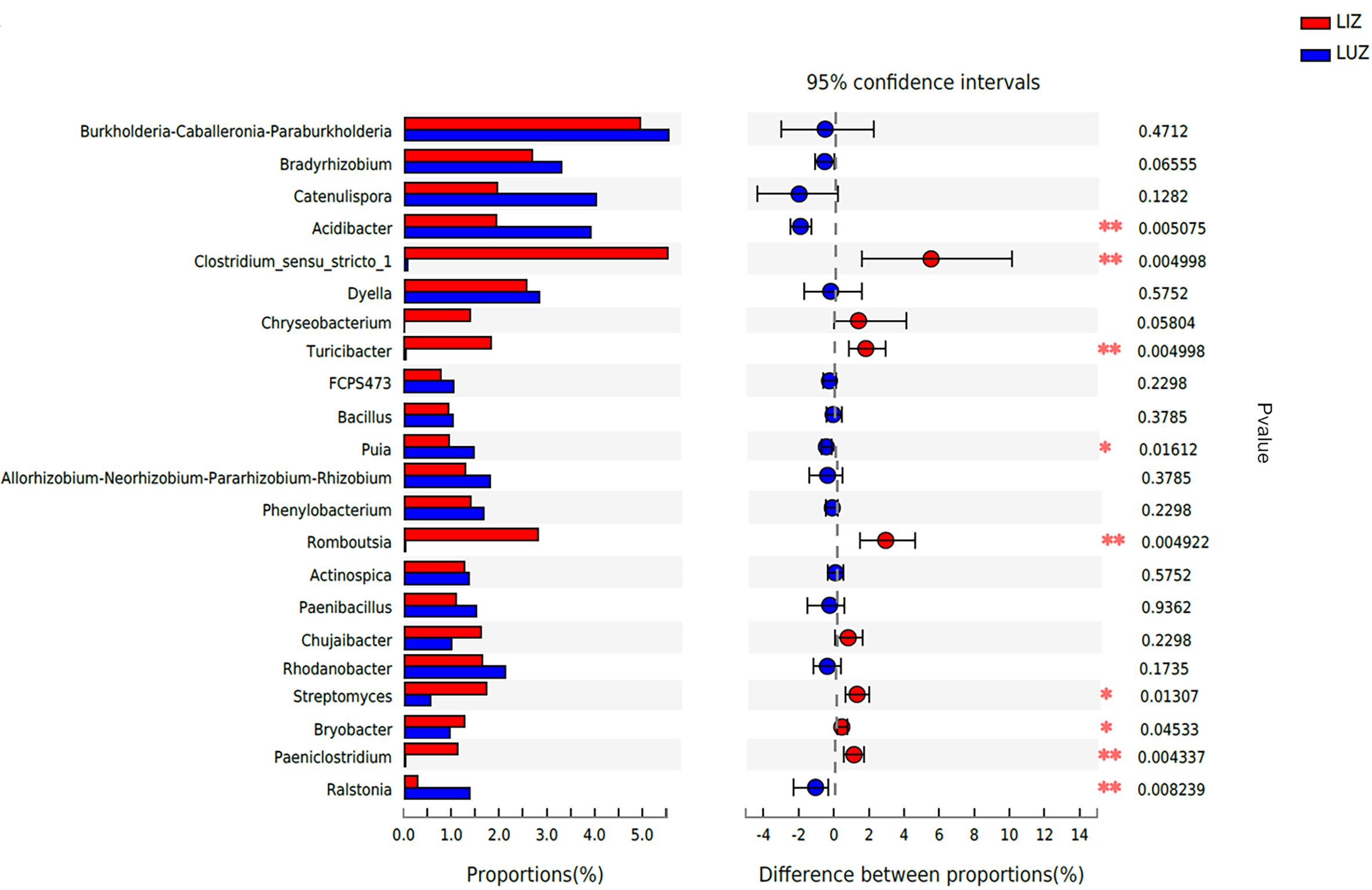

Supplement: Supplementary file 4 — Supplementary Figure S3. [file 41598_2022_6789_MOESM4_ESM.tif]

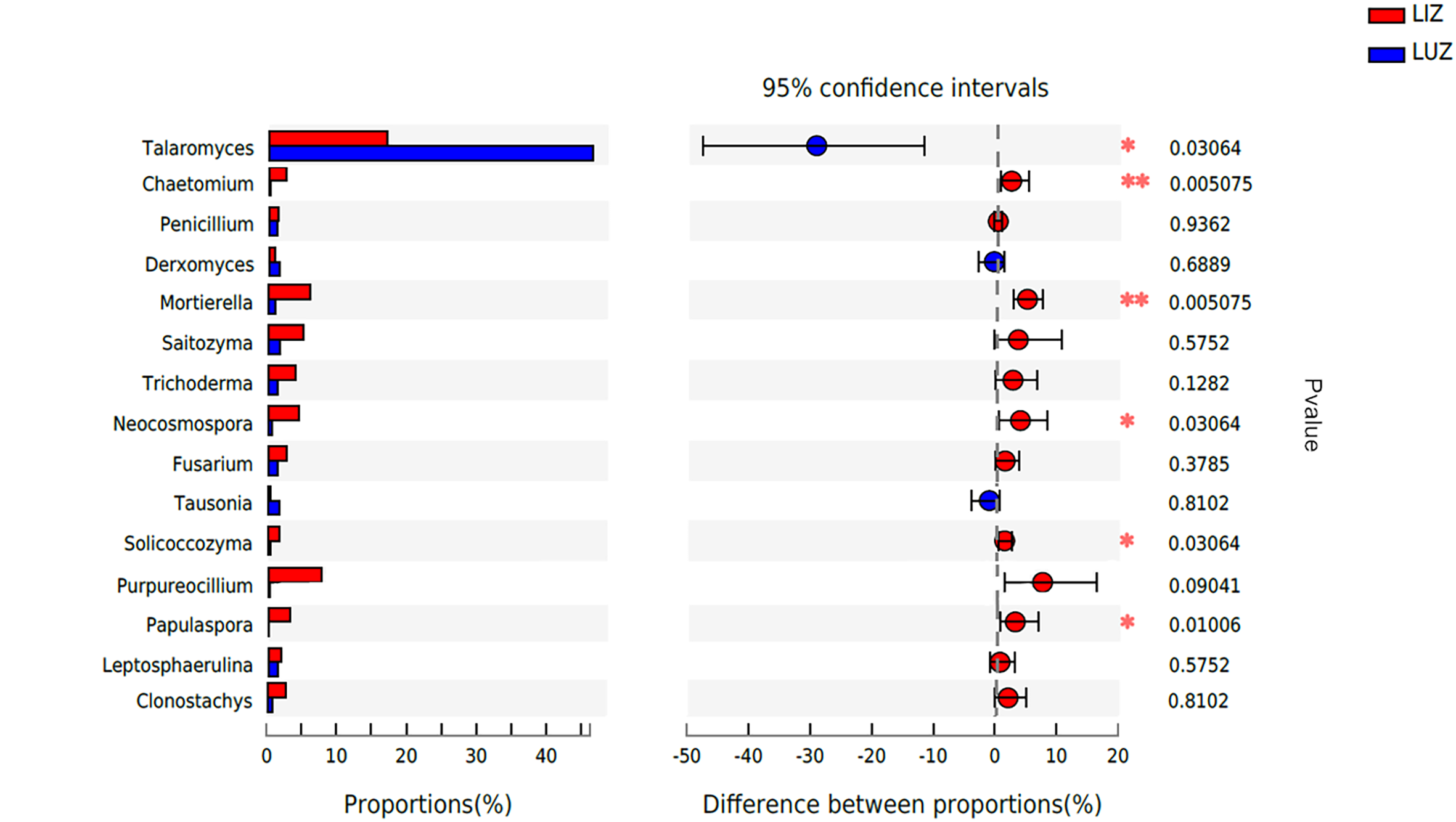

Supplement: Supplementary file 5 — Supplementary Figure S4. [file 41598_2022_6789_MOESM5_ESM.tif]
