# Supplementary material for: Impacts of continuous and rotational cropping practices on soil chemical properties and microbial communities during peanut cultivation
Source: Sci Rep. 2022 Feb 17;12:2758. doi: 10.1038/s41598-022-06789-1 (PMC8854431; doi:10.1038/s41598-022-06789-1)
Supplement: Supplementary file 6 — Supplementary Table S1. [file 41598_2022_6789_MOESM6_ESM.docx]

| Pathogenic fungus | LUZ | LIZ |
| --- | --- | --- |
| *Fusarium* | 1.40% | 2.50% |
| *Penicillium* | 1.18% | 1.22% |
| *Athelia* | 0 | 0.0916% |
| *Gibberella* | 0.0223% | 0.0738% |
| *Colletotrichum* | 0.0509% | 0.065% |
| *Exophiala* | 0.0128% | 0.054% |
| *Nigrospora* | 0.0123% | 0.0203% |

**Table S1.** Relative abundance of pathogenic fungal in two peanut fields at genus level.
